# Selection of suitable reference genes for gene expression analysis in gills and liver of fish under field pollution conditions

**DOI:** 10.1038/s41598-019-40196-3

**Published:** 2019-03-05

**Authors:** Noemí Rojas-Hernandez, David Véliz, Caren Vega-Retter

**Affiliations:** 1 Departamento de Ciencias Ecológicas, Instituto de Ecología y Biodiversidad (IEB), Facultad de Ciencias, Universidad de Chile, Santiago, Chile; 2 Núcleo Milenio de Ecología y Manejo Sustentable de Islas Oceánicas (ESMOI), Departamento de Biología Marina, Universidad Católica del Norte, Coquimbo, Chile

## Abstract

To understand the role of gene expression in adaptive variation, it is necessary to examine expression variation in an ecological context. Quantitative real-time PCR (qPCR) is considered the most accurate and reliable technique to measure gene expression and to validate the data obtained by RNA-seq; however, accurate normalization is crucial. In Chile, the freshwater silverside fish *Basilichthys microlepidotus* inhabits both polluted and nonpolluted areas, showing differential gene expression related to pollution. In this study, we infer the stability of six potential reference genes (tubulin alpha, hypoxanthine-guanine phosphoribosyltransferase, glyceraldehyde-3-phosphate dehydrogenase, beta-actin, 60S ribosomal protein L13, and 60S ribosomal protein L8) in the gills and liver of silverside individuals inhabiting polluted and nonpolluted areas. To validate the reference genes selected, the most and least stable reference genes were used to normalize two target transcripts, one for each organ. The RefFinder tool was used to analyze and identify the most stably expressed genes. The 60S ribosomal protein L8 gene was ranked as the most stable gene for both organs. Our results show that reference gene selection influences the detection of differences in the expression levels of target genes in different organs and, also highlighting candidate reference genes that could be used in field studies.

## Introduction

Water pollution is one of the main threats to global freshwater biodiversity that, together with other human interventions, has impacted freshwater species worldwide^1,2^. One of the described effects of water pollution is a change in gene expression in freshwater organisms. For example, altered gene expression related to DNA replication, response to DNA damage, and cell signaling was found in the freshwater fishes *Dalia pectoralis* and *Pungitius pungitius* inhabiting areas with polychlorinated biphenyls (PCBs)^3^, while Bertucci *et al*. detected differential expression related to translation, response to stimulus, apoptosis, immune response and transport pathways in individuals of the freshwater mussel *Margaritifera margaritifera* exposed to trace metals^4^.

The study of variation in gene expression is fundamental to understanding how organisms respond to environmental changes, such as novel stressors^5–7^. Whereas studies have largely focused on the dynamics of gene expression of organisms under controlled laboratory conditions, few studies have addressed the question of how organisms respond in an ecological context^8^. Whitehead and Crawford argued for the necessity of examining expression variation in ecological contexts to understand the role of gene expression in adaptive variation^6^. Under natural conditions, individuals are exposed to multiple and changing environmental signals, thus providing an opportunity to determine gene expression patterns that cannot be discovered under laboratory conditions^9,10^.

Quantitative real-time PCR (qPCR) is considered the most accurate and reliable technique to measure gene expression and to validate the data obtained by microarrays and RNA-Seq^11^. This is an extremely sensitive method that allows the detection of small changes in gene expression, and thus accurate normalization is a crucial step to correct inevitable experimental variations, such as differences in the amount of starting material^12^ and/or sample loading^13^. For this, one or several reference genes with stable expression across the treatments and/or developmental stages under investigation must be used^14^. Accordingly, the gene expression stability of the reference genes must be verified for each research condition^15^.

In Chile, the Maipo River basin is one of the most disturbed and polluted water bodies, mainly due to the high human population (almost 40% of the Chilean population inhabits this area) and the high concentration of industries^16^. This basin has experienced water quality degradation and eutrophication^17^, mostly as a result of organic matter from untreated sewage. A study of fish diversity in this basin showed a significant reduction in species richness and abundance^18^, likely associated with habitat degradation and pollution. The endemic silverside *Basilichthys microlepidotus* inhabits this basin, although it has decreased significantly in abundance and is currently considered as a “vulnerable” species^19^. Previous studies have described silverside populations inhabiting both nonpolluted and polluted areas of this basin^20^. Furthermore, Vega-Retter *et al*. described candidate loci under directional selection related to the pollution^21^ and found differential gene expression when comparing individuals inhabiting polluted and nonpolluted areas, with the differential gene expression relating to apoptotic processes and cellular proliferation as well as the suppression and progression of tumors^22^. Thus, this system allows for the description of genes with stable expression profiles in a complex and dynamic environment where multiple and simultaneous stimuli occur, providing insights into genes that can be used as reference genes in ecological contexts.

Considering the biological relevance of the results found in the first RNA-seq experiments performed by Vega-Retter *et al*.^22^, it is imperative to determine the stability of this pattern over time and to confirm this pattern with a more accurate technique such as qPCR. Thus, the objective of this study was to assess the gene expression stability of six potential reference genes in individuals of *B. microlepidotus* inhabiting different environmental pollution conditions. For this purpose, we used the geNorm, NormFinder, BestKeeper and the ΔCt algorithms. Considering that each organ could respond differently when faced with pollution^23^, reference genes were identified in two tissues, the gills and liver; the first exposed directly to the pollution through the water and the second by food^24,25^, accumulating different amounts of pollutants (e.g., metals) as described by Copaja *et al*.^26^ for our study species. To validate the selected reference genes, one target gene for each organ was used. The ring finger protein 19B (*rnf19b*) gene was used as a target for the gills since it is involved in crucial molecular mechanisms, such as the molecular function of metal ion binding, and because it participates in the adaptive immune response. For the liver, the mitogen-activated protein kinase kinase kinase 5 (*map3k5)* gene was targeted to validate the candidate reference genes. *The map3k5* gene is also involved in metal ion binding and in biological processes, such as innate immune response, positive regulation of apoptotic process, cellular response to tumor necrosis factor and positive regulation of transcription, DNA-templated.

## Results

### Selection of candidate reference genes and PCR efficiency

**Figure 1:**
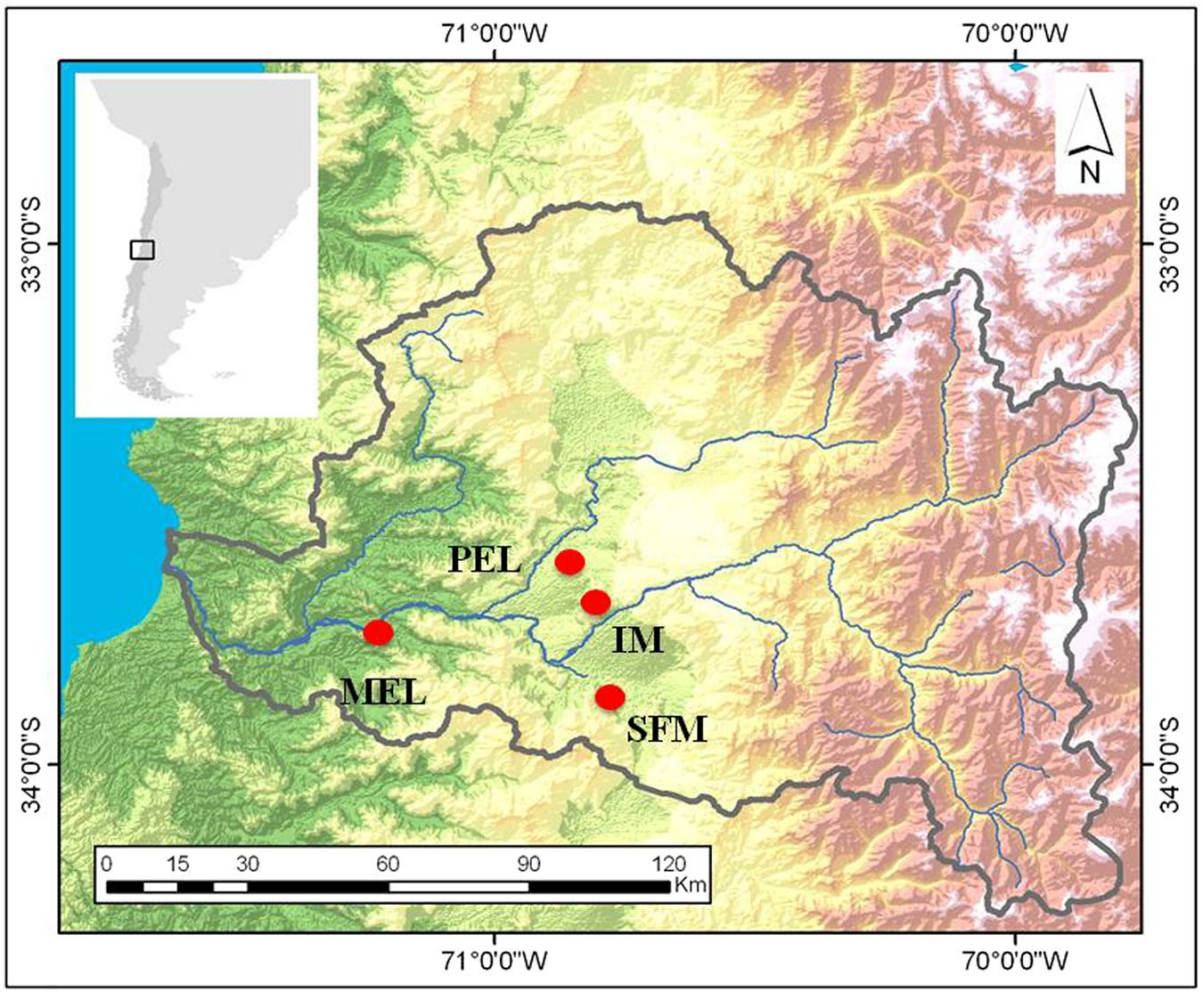
Sampling sites of *Basilichthys microlepidotus* in the Maipo River basin. MEL = Melipilla; PEL = Pelvin; IM = Isla de Maipo; SFM = San Francisco de Mostazal.

Considering the information obtained from the literature regarding reference genes in fishes, we designed a set of six candidate reference genes, namely, hypoxanthine-guanine phosphoribosyltransferase (*hprt*), beta-actin (*actb*), glyceraldehyde-3-phosphate dehydrogenase (*gapdh*), 60S ribosomal protein L8 (*rpl8*), tubulin alpha (*tuba*) and 60S ribosomal protein L13 (*rpl13*) (Table 1), which we then tested in fish collected from polluted (Isla de Maipo: IM, Pelvin: PEL, Melipilla: MEL) and nonpolluted (San Francisco de Mostazal: SFM) sites (Fig. 1; Table 2). Primer specificity was verified by melting curve analysis for each gene. PCR efficiency values varied between 96.6 and 100.6%, and correlation coefficient values (R^2^) ranged from 0.994 to 0.999 across all primer pairs (Table 1).

**Table 1:** Summary of selected primers in order to be tested like candidate reference genes in *Basilichthys microlepidotus* and their parameters derived from qPCR analysis. R^2^: correlation coefficient.

Gene Symbol	Gene Name	Accession number	Sequence primer 5′-3′	Size (bp)	PCR efficiency (%)	R^2^
*tuba*	Tubulin alpha	MH886397	F:CATTGACGAAGTGCGAACCG R:TTAGCCGCATCCTCCTTTCC	81	99.939	0.999
*hprt*	Hypoxanthine-guanine phosphoribosyltransferase	MH886398	F:CGACACAGGGAAGACGATGA R:GACCTCAAATCCTACAAAGTCCG	139	98.993	0.996
*gapdh*	Glyceraldehyde-3-phosphate dehydrogenase	MH886399	F:CAGCGTGTGGTTGTGTCTG R:GTACAGGAGGCATTACTGACG	113	100.039	0.999
*actb*	Beta-actin	MH886400	F:AGGAGATGGGAACTGCTGC R:CCATACCAAGGAAGGAAGGCT	135	98.675	0.999
*rpl13*	60S ribosomal protein L13	MH886401	F:CCAACGTGCAGCGGCCTGAA R:CGTGGCCATCTTGAGTTCCT	111	100.597	0.994
*rpl8*	60S ribosomal protein L8	MH886402	F:CCGTCGTGGGTGTGGTC R:CAGCAGTTCCTCTTGGCCTT	97	96.635	0.998
*rnf19b*	Ring finger protein 19B	MH886403	F:AAGTTCGTCAACAGACCCGA R:CTGATCCAGCAGGCTACGAC	138	105.826	0.994
*map3k5*	Mitogen-activated protein kinase kinase kinase 5	MH886404	F:CGCAAAGTGGGAGTTAAGCTG R:CTGGACGACCCTGGTTTTGT	132	100.043	0.98

**Table 2:** Description of the study sites, location, British Columbia Water Quality Index (BCWQI) category and pollution condition.

Site	Location	BCWQI category	Condition
San Francisco de Mostazal (SFM)	33°58′19.97″S; 70°42′56.49″O	Acceptable	Nonpolluted
Isla de Maipo (IM)	33°44′58″ S; 70°53′26″O	Poor	Polluted
Pelvin (PEL)	33°36′21″S; 70°54′33″O	Marginal	Polluted
Melipilla (MEL)	33°42′50″S; 71°12′39″O	Marginal	Polluted

**Figure 2:**
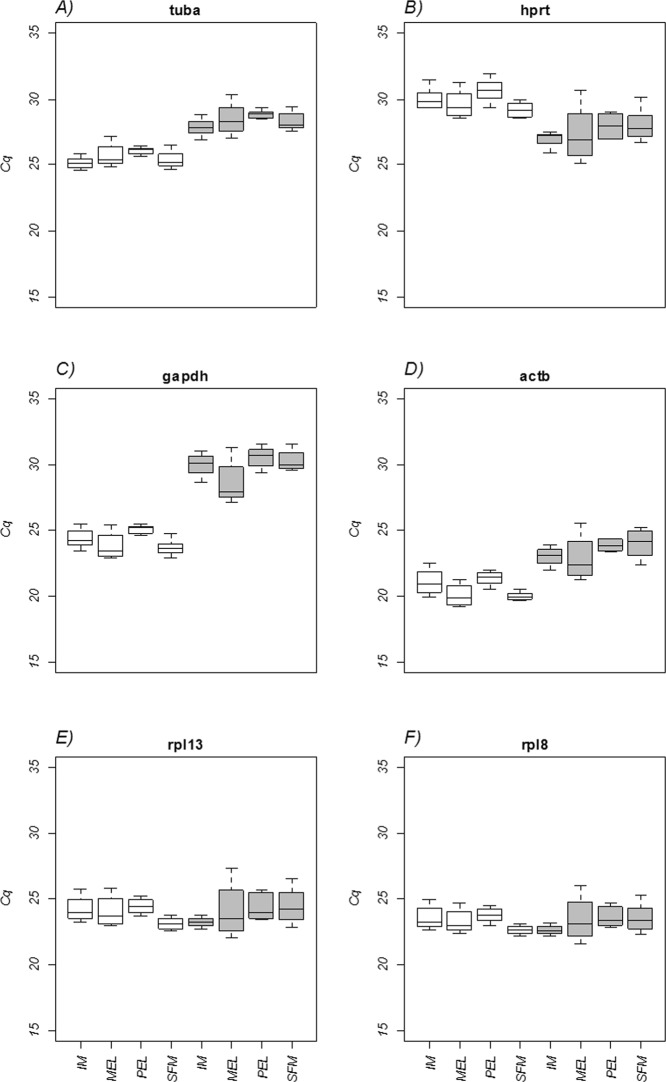
Quantification cycle (Cq) values for the six candidate reference genes for each sample site. White boxes represent gill tissue, and gray boxes represent liver tissue.

The expression of the six candidate genes was examined in gill and liver tissues (Fig. 2). The lowest expressed reference gene in the gill tissue was *hprt* (mean Cq = 29.88, SD = 0.95), while *actb* showed the highest expression level (mean Cq = 20.63, SD = 0.92). In liver, *gapdh* had the lowest expression level (mean Cq = 29.75, SD = 1.30), whereas *actb* and *rpl8* exhibited the highest gene expression (mean Cq = 23.55, SD = 1.19 and mean Cq = 23.53, SD = 1.14, respectively). Additionally, *tuba* was the least variable candidate gene, with a coefficient of variation (CV) of 2.65%, and *actb* showed the highest variability, with CV = 4.44%, in gill tissue. In liver, *tuba* was the least variable gene (CV = 2.93%), while *rpl8* was most variable (CV = 5.66%).

### Analysis of gene expression stability in gill tissue

The stability of expression levels across environmental conditions was determined for each sample tissue using the four methods (geNorm, NormFinder, ΔCt and BestKeeper) implemented in the RefFinder web tool. Particularly in gill tissue, *rpl8* and *hprt* ranked as the most stable genes under the geNorm, NormFinder and ΔCt methods, while the BestKeeper algorithm ranked *tuba* and *rpl8* as the most stable genes. Using the geometric mean of ranked values from all methods, RefFinder generated a comprehensive ranking in which *rpl8* and *hprt* were the most stable candidate reference genes for gill tissue, while *actb* was the least stable gene (Table 3). To determine the number of reference genes required for reliable normalization, the pairwise variation (V) was calculated using the geNorm method. The gill tissue data showed a V_2/3_ < 0.15 (Supplementary Fig. S1), indicating that two reference genes are the optimal number and that no additional genes are needed for normalization.

**Table 3:** Stability of candidate reference genes in gill tissue.

Gene	geNorm		NormFinder		Best Keeper		Delta Ct		Comprehensive ranking	
	M value	Rank	Stability value	Rank	SD [+/−CP]	Rank	Average of standard deviations	Rank	Geometric mean of ranking values	Rank
*rpl8*	0.192	1	0.188	1	0.67	2	0.4	1	1.19	1
*hprt*	0.316	2	0.261	2	0.79	6	0.44	2	2.91	2
*rpl13*	0.192	1	0.32	4	0.77	5	0.46	4	2.99	3
*gapdh*	0.398	4	0.287	3	0.75	3	0.46	3	3.41	4
*tuba*	0.481	5	0.584	6	0.57	1	0.65	6	3.83	5
*actb*	0.383	3	0.36	5	0.77	4	0.48	5	4.47	6

### Analysis of gene expression stability in liver tissue

The geNorm, NormFinder and BestKeeper methods showed that the *rpl8* gene is the most stable gene; however, different genes were ranked as the second most stable gene. Further, the ΔCt method ranked the *rpl13* gene in first position, followed by the *hprt* gene. A comprehensive ranking of the four algorithms obtained with the RefFinder tool showed that *rpl8* and *rpl13* were the most stable candidate reference genes for liver tissue, while *gapdh* was the least stable gene (Table 4). Additionally, the pairwise variation (V) values obtained using the geNorm method showed that two reference genes are recommended for liver tissue (V_2/3_ < 0.15) (see Supplementary Fig. S2).

**Table 4:** Stability of candidate reference genes in liver tissue.

Gene	geNorm		NormFinder		Best Keeper		Delta Ct		Comprehensive ranking	
	M value	Rank	Stability value	Rank	SD [+/−CP]	Rank	Average of standard deviations	Rank	Geometric mean of ranking values	Rank
*rpl8*	0.308	1	0.203	1	0.85	1	2.06	3	1.32	1
*rpl13*	0.308	1	0.433	4	1.04	4	2.05	1	2	2
*hprt*	0.354	2	0.306	2	0.98	3	2.06	2	2.45	3
*actb*	0.45	3	0.368	3	0.95	2	2.13	4	3.13	4
*tuba*	1.954	4	4.193	5	1.42	5	4.64	5	5	5
*gapdh*	3.015	5	4.814	6	1.9	6	5.14	6	6	6

### Validation of the selected reference genes

For the validation of the reference genes selected in gills and liver, different normalizations were applied, the three most stable normalizations: (i) the most stable gene, (ii) the second most stable gene, (iii) the first and second most stable genes and, the two less stable normalizations: (i) the least stable gene and (ii) the two least stable genes.

### Gills

**Figure 3:**
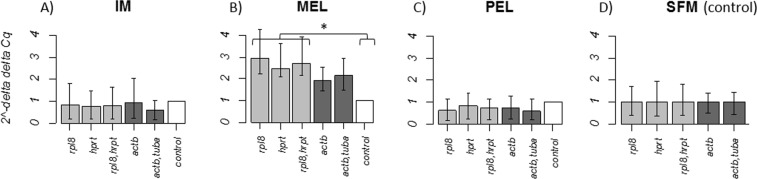
Expression level of the *rnf19b* gene in gill tissue normalized by the most stable reference genes (*rpl8* and *hprt*) in comparison to normalization with the least stable reference genes (*actb* and *tuba*). (**A**) Relative expression of *rnf19b* in individuals from site IM, (**B**) individuals from sites MEL, (**C**) PEL and (**D**) SFM. Control bar represents the relative expression of the calibrator (site SFM). Whisker caps represent the minimum and maximum values of the 2^−ΔΔCq^. *P < 0.05.

The choice of reference genes in gill tissue was validated with the analysis of relative expression of the *rnf19b* gene. IM site did not show significant differences in the expression level but it was observed an increase in the expression level when the *rnf19b* gene was normalized by the least stable gene (*actb*) while, a not significant decrease was observed when it was normalized by the two least stable reference genes (*actb*, *tuba*) (Fig. 3A). However, when the expression levels of the three most stable normalizations were compared with the expression levels of the two less stable normalizations no significant differences were found (*P* > 0,05). In the case of the IM site, for all of the normalizations used, the individuals showed expression levels close to the control group. At the MEL site, the expression levels of *rnf19b* for all normalizations were higher than those in the control group, but significant differences were only found between the three most stable normalizations and the control group (*P* = 0.01), not between the least stable normalizations and the control group (*P* = 0.07) or between the most and least stable reference genes (*P* = 0.09) (Fig. 3B). At the PEL site, the expression level of *rnf19b* was close to that of the control group, and no significance differences were detected between the normalizations or with the control group (Fig. 3C).

### Liver

**Figure 4:**
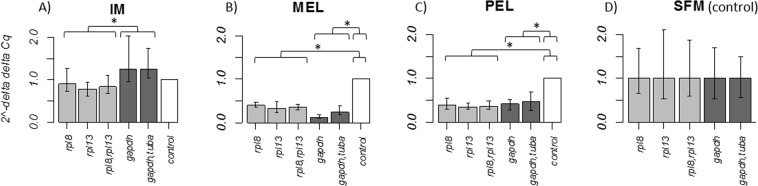
Expression level in liver tissue of the *map3k5* gene normalized by the most stable reference genes (*rpl8* and *rpl13*) in comparison to normalization with the least stable reference genes (*gapdh* and *tuba*). (**A**) Relative expression of *map3k5* in individuals from site IM, (**B**) individuals from sites MEL, (**C**) PEL and (**D**) SFM. Control bar represents the relative expression of the calibrator (site SFM). Whisker caps represent the minimum and maximum values of the 2^−ΔΔCq^. *P < 0.05.

The expression profile of the *map3k5* gene was analyzed to validate the selected reference genes. The expression levels of the *map3k5* gene in fish collected at the IM site compared to the fish collected at the SFM site (control group) were similar when the three most stable normalizations were applied (Fig. 4A). However, when the *map3k5* gene was normalized by the least stable (*gapdh*) and the two least stable reference genes (*gapdh*, *tuba*), the expression levels of *map3k5* increased compared to the control group, however this increase was not statistically significant. Only the comparison between the three most stable and the two least stable normalizations showed significant differences (*P* < 0.05) (Fig. 4A). For all the normalizations used the expression level of the *map3k5* gene in fish collected at the MEL site was lower when compared to the control group, finding significant differences of the three most stable normalizations (*P* = 0.004) and the two least stable normalization (*P* = 0.002) with the control group. However, no significant differences were found between the two types of normalization (*P* = 0.07) (Fig. 4B). In the case of PEL, no significant difference was detected between the expression level obtained with the three most stable ant the two least stable normalizations (*P* = 0.18). However, both kinds of normalizations were significantly different with respect to the control group (*P* = 0.0008 and *P* = 0.001 for normalization with the three most stable and two least stable normalizations, respectively) (Fig. 4C).

## Discussion

It is well known that accurate normalization is necessary to obtain reliable results^15^, and for this reason, the selection and validation of reference genes should be realized for each experimental or environmental condition. For *B. microlepidotus*, however, no reference genes have been described. We selected six reference genes commonly used in fish and evaluated which of them exhibit stable expression across all sites for gill and liver tissue. Three of the four algorithms used and the RefFinder comprehensive ranking tool ranked *rpl8* as the most stable gene for both organs, making it a good candidate reference gene under uncontrolled or highly variable conditions, such as field studies. The second and third most stable genes were *hprt* and *rpl13* for the gill tissue, respectively, while for the liver, *rpl13* is the second most stable gene, and *hprt* is the third. Therefore, both of these genes also appear as two potential candidate reference genes for field studies.

Interestingly, two ribosomal proteins (*rpl8* and *rpl13*) were within the three most stable genes in both organs. In other studies, ribosomal proteins have been selected as reference genes for gill or liver tissue in zebrafish in response to various stimuli, such as exposure to cadmium chloride and subsequent infection by bacteria^27^, glyphosate-based herbicide exposure^28^ and response to arsenic^29^.

Moreover, to determine the influence of different reference genes in normalization, one target gene per tissue was evaluated. The results obtained demonstrated the importance of reference gene selection: (i) significant expression level differences were recovered for the target transcripts when the most and least stables genes were used to normalize and, (ii) significant differences were found with respect to the control when normalizing with the most stable genes that were not detected when normalizing with the least stable genes. These types of errors in gene expression-level estimations can lead to serious and dangerous biological misinterpretations.

This is the first study to analyze and validate reference genes for the Chilean silverside *B. microlepidotus*, which inhabits areas with different degrees of pollution and, as far we know, is one of the few studies evaluating the performance of six widely used reference genes in wild conditions where individuals are exposed to multiple factors that simultaneously change among the treatments (sites) studied. It is important to note that the environmental conditions are changing in a field study thus it is necessary to test the candidate genes in each new study, even if the study is performed in the same geographical sites. Overall, we consider that this is an important topic to consider in field studies due to the high intra- and inter-annual variation in the environmental condition.

This work not only shows the importance of reference gene selection, noting its influence on the detection of significant differences between expression levels and therefore in the robustness of the biological conclusions, it also suggests reference genes that could work under field conditions even in different organs.

## Methods

### Sample Collection

Vega-Retter *et al*. described five independent populations of the silverside *Basilichthys microlepidotus* within the Maipo River basin^20^. Four of these populations were chosen for this new research, one nonpolluted (NP) site, San Francisco de Mostazal (SFM), and two polluted (P) sites, Pelvin (PEL) and Melipilla (MEL) (Fig. 1). It is important to note that the fourth site, Isla de Maipo (IM), has changed from nonpolluted in previous studies^20–22^ to polluted in the present study. The data obtained in 2016 and 2017 pointed out that IM has suffered considerable water quality degradation, with its physicochemical characteristics close to MEL and PEL (unpublished data). Thus, we classified IM as a polluted site for this study. From each site, six individuals of *Basilichthys microlepidotus* were collected from November 2016 to January 2017. Livers and gills were dissected out and snap-frozen by immersion in liquid nitrogen. The tissues were then stored at −80 °C for subsequent RNA extraction. Four individuals per site were chosen for the selection and validation of reference genes, except for liver in the PEL site where some samples reached low RNA quantity after extraction, thus only three individuals could be used. All the individuals used were adults showing average lengths of: SFM_gills and liver_ = 13.23 cm (0.95), IM_gills and liver_ = 8.53 cm (0.39), MEL_gills and liver_ = 6.1 cm (0.49), PEL_gills_ = 8.63 cm (1.36) and PEL_liver_ = 8.23 cm (1.27); standard deviation is show in parenthesis.

All of the protocols of this study were approved by the ethics committee of the Universidad de Chile and complied with existing laws in Chile (Resolución Exenta No. 3078 Subsecretaria de Pesca).

### RNA isolation and cDNA synthesis

Total RNA was isolated from the gills and liver tissues using the PureLink RNA Mini Kit (Invitrogen, United States) and treated with the TURBO DNA-free kit (Invitrogen) to remove gDNA, following the manufacturer’s instructions in both cases. RNA concentration was measured using a Qubit RNA BR Assay Kit (Invitrogen), and its quality was evaluated with an Agilent 2100 Bioanalyzer (Agilent Technologies). Only samples showing an RIN > 6.5 were selected for the following analysis. For each sample, first-strand cDNA was synthesized from 1 µg of total RNA with the Verso cDNA Synthesis Kit (Thermo Scientific) following the manufacturer’s instructions. No reverse transcriptase controls were included to assess the presence of gDNA contamination. Finally, cDNA was diluted 10 fold for qPCR analysis.

### Screening of candidate reference genes and primer design

An extensive bibliographic search was performed to identify the reference genes widely used in fish. From these candidate reference genes obtained through the literature and for the target genes, primers were designed with the Primer-BLAST tool (https://www.ncbi.nlm.nih.gov/tools/primer-blast/) using sequences obtained from RNA-seq of *B. microlepidotus* (unpublished data). All primers were designed using the following settings: amplicon length between 80 and 150 bp; melting temperature ranging from 57 to 63 °C with an optimal Tm of 60 °C; GC content ranging between 40 and 70% and primer size between 15 and 25 base pairs. Before qPCR, the specificity of the primers was confirmed by conventional PCR and by loading amplicons onto a 1.5% agarose gel. Also, the three most stable genes were sequencing to confirm the primer specificity (Supplementary Table S1).

### qPCR

For qPCR assays, a StepOne Real Time PCR System (Applied Biosystems, Foster City USA) was used. Quantitative PCR were performed using 2 µL of diluted cDNA (70 ng/uL), 10 µL of Fast SYBR Green Master Mix (Applied Biosystems), 0.4 µL of each primer in 20 µL of reaction mixture and for 20 s at 95 °C, followed by 40 cycles of 95 °C for 3 s and 60 °C for 30 s. At the end of each reaction, melting curves were evaluated to verify primer specificity. A cDNA pool was prepared, and a dilution series (factor 1/5) was performed to generate a standard curve for each primer pair. The PCR amplification efficiency was estimated using the StepOne Software v2.3 (Applied Biosystems). The experiments were performed using four biological samples for each study site and tissue, three technical replicates for each sample and the no reverse transcriptase control in duplicate. In each plate, a no-template control was included.

### Data Analysis

Because the qPCR efficiencies varied slightly with respect to the optimum, we used noncorrected Cq values to calculate the stability of the candidate reference genes from different environmental conditions using the geNorm, NormFinder, BestKeeper and ΔCt algorithms, all implemented in the RefFinder platform. This web tool assigns a score to each gene, considering the ranking obtained from each algorithm, and calculates the geometric mean of their scores to establish a comprehensive stability ranking^30^.

To identify the optimal number of reference genes required for normalization, we used the method described by Vandesompele *et al*.^31^ and implemented in the geNorm module inside qbase+ software^32^. This method calculates pairwise variation values (V = V_n/n+1_) between the two sequential normalization factors (NF_n_ and NF_n+1_) for all samples within the same tissue. Large variation means that the added gene has a significant effect and should preferably be included for the calculation of a reliable normalization factor. A cut-off value of 0.15 was used, below which the inclusion of an additional control gene is not required.

### Reference Gene Validation

To determine whether the reference genes selected affect the normalization of our genes of interest, we analyzed the relative expression of two target genes. *rnf19b* was amplified in gill tissue, while *map3k5* was amplified in liver tissue. The two most and two least stable reference genes were used to normalize the target gene expressions according to the 2^−ΔΔCt^ method^33^ using the library NormqPCR^34^ implemented in R^35^. Individuals from SFM were used as a control group. The gene expression levels of the most stable normalizations (normalizations used: the most stable gene, the second most stable gene and the first and second most stable genes), the least stable normalizations (normalizations used: the least stable gene and the two least stable genes) and the expression level of the control group were compared using permutation tests implemented in the library lmPerm^36^ in R.

## Supplementary information

Supplementary Information

## Data Availability

Primer sequences: GenBank accession numbers MH886397 - MH886404.
