# Supplementary material for: Selection of suitable reference genes for gene expression analysis in gills and liver of fish under field pollution conditions
Source: Sci Rep. 2019 Mar 5;9:3459. doi: 10.1038/s41598-019-40196-3 (PMC6401100; doi:10.1038/s41598-019-40196-3)
Supplement: Supplementary file 1 — Supplementary Information [file 41598_2019_40196_MOESM1_ESM.pdf]

Selection of suitable reference genes for gene expression analysis in gills and liver of fish under field pollution conditions

Noemí Rojas-Hernandez[1], David Véliz[1,2], Caren Vega-Retter[1]

Supplementary Table S1: sequences of the three most stable genes: 60S ribosomal protein L8 (*rpl8*); 60S ribosomal protein L13 (*rpl13*); Hypoxanthine-guanine phosphoribosyltransferase (*hprt*).

Gene	Sequence
rpl8	ATCCTGAATGCCGGTCGTGCCTACCACAAGTACAAGGCCAAGAGGAACTGCTG
rpl13	CAACGTGCAGCGGCCTGAAGGAGTACCGCTCCAAGCTCATCCTGTTCCCCCAG AAAAGGCTTCTGCACCCAAGAAGGGAGACAGCTCTGAGGAGGAACTCAAGAT GGCCACGA
hprt	TGGTTAAAGTAGCAAGTTTGCTGGTGAAGAGAACGCCGCGAAGCGTCGGCTAC CGACCGGACTTTGTAGGATTTGAGGTCG

Figures:

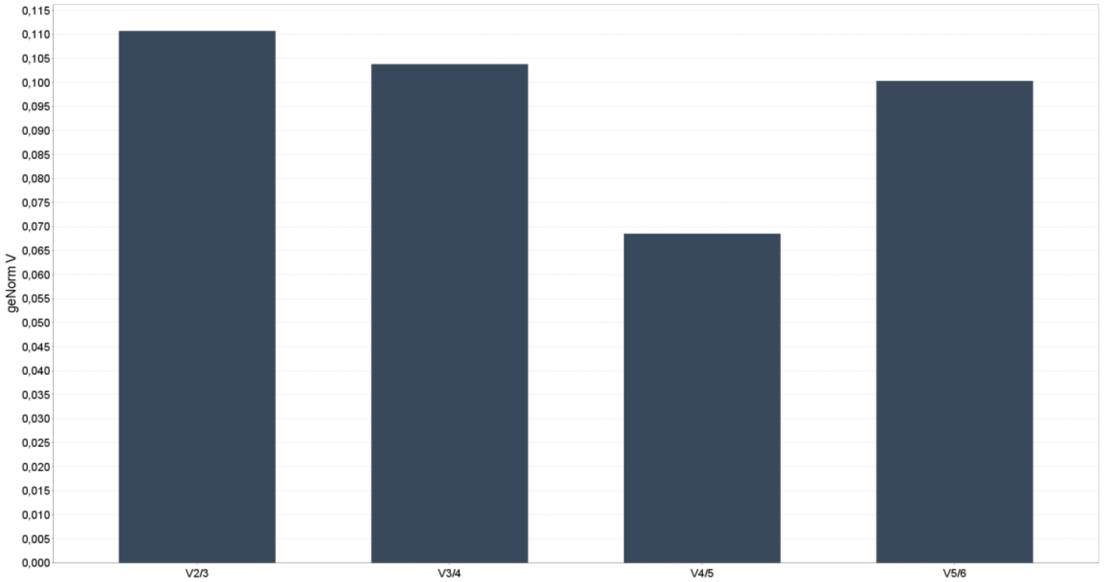

Supplementary Figure S1. Pairwise variation values (geNorm V) versus the increment in number of reference genes for gill tissue.

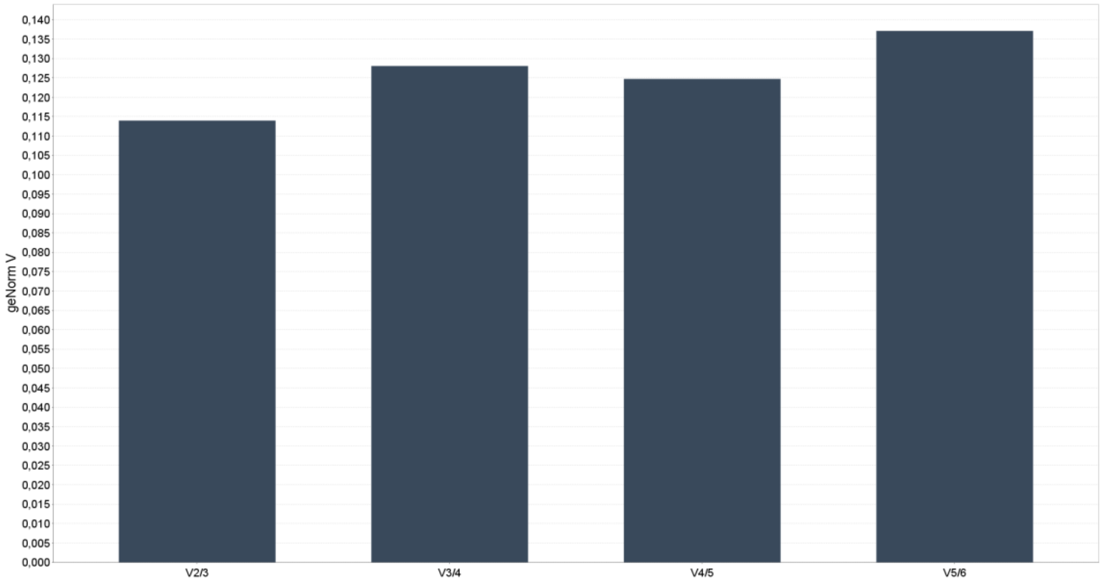

Supplementary Figure S2. Pairwise variation values (geNorm V) versus the increment in number of reference genes for liver tissue.
